# Short-Term Effects of an eHealth Care Experiential Learning Program Among Patients With Type 2 Diabetes: Randomized Controlled Trial

**DOI:** 10.2196/53509

**Published:** 2024-08-16

**Authors:** Yu-Shan Cheng, Cheng-Pei Lin, Lu-Yen Anny Chen, Wei-Ren Hwang, Yi-Chun Lin, Yu-Chi Chen

**Affiliations:** 1 School of Nursing The University of Texas at Austin Austin, TX United States; 2 Institute of Community Health Care College of Nursing National Yang Ming Chiao Tung University Taipei Taiwan; 3 Cicely Saunders Institute of Palliative Care, Policy and Rehabilitation King's College London London United Kingdom; 4 Institute of Clinical Nursing College of Nursing National Yang Ming Chiao Tung University Taipei Taiwan; 5 Rong-Yang Clinic Taipei Taiwan; 6 Division of Endocrinology and Metabolism Department of Medicine Taipei Veterans General Hospital Taipei Taiwan

**Keywords:** diabetes, eHealth literacy, eHealth, patient engagement, experiential learning theory, experimental learning theory, type 2 diabetes, randomized controlled trial

## Abstract

**Background:**

Type 2 diabetes is a chronic disease with a significant medical burden. eHealth care integrates medicine and technology to enhance the outcomes of such patients; however, adequate eHealth literacy (eHL) is necessary for that to happen. Fostering eHL is crucial for patients with diabetes to engage with eHealth care and receive quality care and timely support. Experiential learning theory can enhance patients’ eHL and skills to use eHealth care technology in their daily care.

**Objective:**

This study explored the effectiveness of an eHealth care experiential learning program in improving eHL, patient health engagement, and eHealth care use status among patients with type 2 diabetes in 3 months.

**Methods:**

In this randomized controlled trial, patients under case management services from various clinics in Taiwan were randomly assigned to either the intervention group receiving the 6-session eHealth care experiential learning program or the control group receiving the usual care. Data were collected using structured questionnaires at 3 time points: pretest, postintervention, and 3 months after the intervention. Descriptive data were presented using frequency distribution, percentage, mean, and SD. The outcomes were analyzed using a generalized estimating equation method by intention-to-treat analysis.

**Results:**

A total of 92 participants (46 in each group) were recruited in this study. Of these, 86 completed the course and follow-up evaluations with a mean age of 62.38 (SD 12.91) years. After completing the intervention, the intervention group had significantly higher posttest scores in eHL (β=19.94, SE 3.52; *P*<.001), patient health engagement (β=.28, SE 0.13; *P*=.04), and eHealth use (β=3.96, SE 0.42; *P*<.001) than the control group. Furthermore, the intervention group maintained these significant improvements in eHL (β=18.19, SE 3.82; *P*<.001) and eHealth use (β=3.87, SE 0.49; *P*<.001) after 3 months.

**Conclusions:**

Participating in the eHealth care experiential learning program resulted in significant improvements in eHL, patient health engagement, and eHealth use among patients with type 2 diabetes. Our interventional program can inform future clinical practice and policies to strengthen self-management skills and facilitate the use of health technology in caring for patients with chronic diseases.

**Trial Registration:**

ClinicalTrials.gov NCT05180604; https://clinicaltrials.gov/ct2/show/NCT05180604

## Introduction

Diabetes is a chronic disease, and its prevalence is rapidly increasing globally. It is currently ranked among the top 10 causes of death worldwide. According to the World Health Organization, over 460 million people have diabetes, with this number expected to reach 700 million by 2045 [1,2]. Diabetes is often accompanied by several complications, leading to soaring medical expenses that impose a heavy burden on patients and their families. In the United States, the cost of direct and indirect care for diabetes is US $327 billion. Moreover, from 2012 to 2017, the economic costs of diabetes increased by 26% in the United States [3]. Therefore, preventing the progression of diabetes is a crucial public health issue.

Diabetes carries significant risks, including pathological changes, irreversible complications, and even death. To effectively manage diabetes, maintain a stable condition, and mitigate these risks, attentive, self-managed daily care by patients is necessary [4]. Patients need to engage in various self-management tasks such as monitoring their blood sugar regularly, managing medication, exercising regularly, eating a healthy diet, and monitoring their condition periodically [5,6]. These interventions can slow disease progression, prevent complications, reduce medical expenses, and enable patients to coexist with the disease [7].

With the rapid development of eHealth technology, using eHealth care systems for chronic disease management has become a growing trend. This innovative strategy allows individuals to efficiently engage in disease self-management in their daily lives and meet continuous care demands. Available applications include those offering physiological monitoring, recordkeeping for self-management at home, health consultation guidance, location and access to emergency services, and communication through social media [8,9]. However, while eHealth applications offer multiple health care solutions, they also experience potential problems, especially if patients are unfamiliar with mobile health or do not know how to use eHealth care systems. In that case, they may not be able to access the service, be apprehensive, or refuse to use such systems altogether. This can result in decreased willingness and motivation to use eHealth care for self-management, leading to deteriorating health outcomes [10,11]. Therefore, improving the engagement of patients with diabetes in the eHealth care system is crucial for continuing to provide them with high-quality care.

eHealth literacy (eHL) refers to an individual’s ability to seek, find, understand, and evaluate health information obtained from electronic sources such as websites, mobile apps, and other digital platforms [12,13]. It involves the skills, knowledge, and capabilities needed to navigate, comprehend, and use digital health resources effectively to make informed decisions on health-related matters. Patients with insufficient eHL find it difficult to use eHealth care applications and respond to their requirements such as completing web-based registration and electronic card check-in, which can result in increased passive resistance to self-management. Conversely, those with higher eHL levels can navigate and operate eHealth care effectively [14-16]. Moreover, enhancing patients’ engagement in self-management of their health is key to promoting their further use of smart health care systems in the eHealth era. Such patient health engagement is a continuous, dynamic process through which patients’ cognition, emotions, and behaviors drive psychological change across the following 4 stages: blackout, arousal, adhesion, and eudaimonic project [17,18]. As a result, patient health engagement affects the health outcomes of chronic disease management.

Introducing smart health care applications could be a beneficial strategy for patients; however, if they cannot engage in smart health care or face serious challenges while using eHealth in their daily care, they may feel reluctant, or even refuse, to use eHealth. Thus, teaching patients to use eHealth care systems and improving their eHL based on their pace may help them engage in eHealth care. In addition, encouraging patients to engage in practical work, discuss with each other, and reflect on their health issues during the learning process can increase their knowledge, attitudes, and behaviors [19,20]. Experiential learning theory (ELT) emphasizes the role of a patient’s experience in developing the skills they need to function in their daily life. This theory has been widely used in education and shown to improve learners’ overall knowledge, attitudes, and behaviors [21-26]. In the eHealth era, it is crucial to improve patients’ eHL to enable them to engage in self-management behaviors. Patients with higher levels of eHL are better able to effectively implement eHealth systems in their daily self-management [27,28], corresponding with the expected achievement indicators of this research. Therefore, we propose that adapting ELT as an intervention for patients’ eHealth care education would enhance their eHL, allow them to engage in smart technology health care, and increase their use of health technology, thus enabling eHealth care to become a part of the lives of patients with diabetes. Ultimately, not only does it enhance self-management behavior, but it can also achieve disease control in the long run, preventing deterioration and complications.

Thus, this study aimed to explore the effectiveness of an eHealth care experiential learning program in improving eHL, patient health engagement, and eHealth care use status among patients with type 2 diabetes.

## Methods

### Overview

This randomized controlled trial reports on the results of a 3-month study that included 92 patients with type 2 diabetes that examined the short-term effects on eHL, patient health engagement, and eHealth care use status. The experimental group received an eHealth care experiential learning program, while the control group received the usual standard care. CONSORT (Consolidated Standards of Reporting Trials) guidelines were used to report the findings (Multimedia Appendix 1).

### Study Design

**Figure 1:**
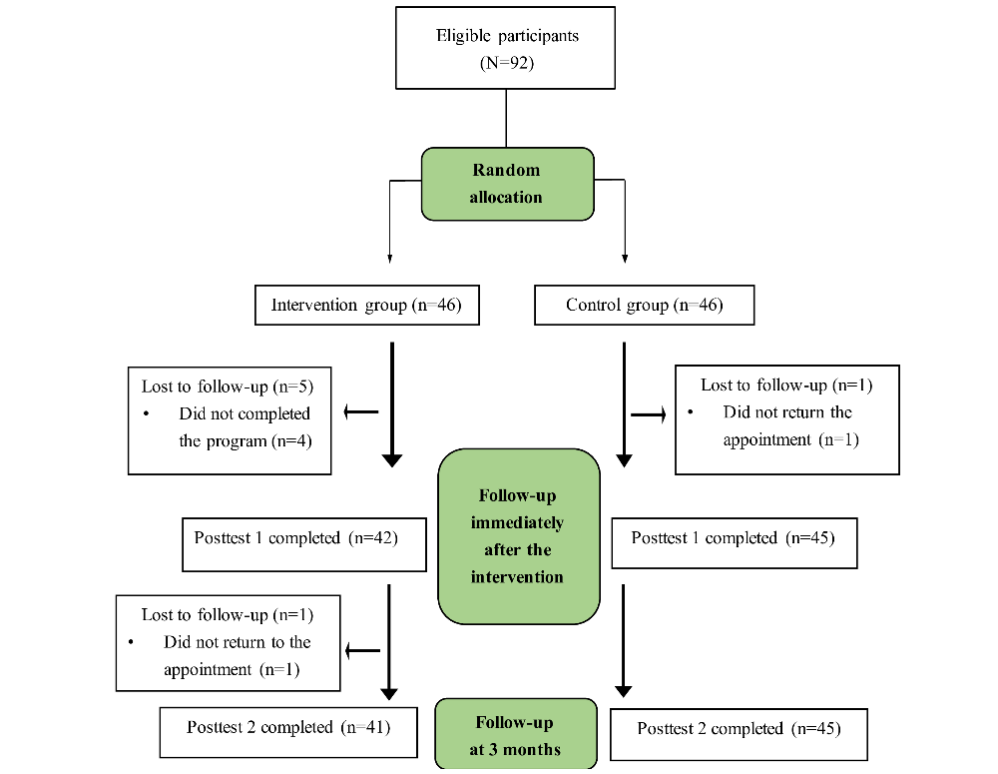
Study process flowchart.

This was a single-blind, randomized controlled trial, in which the research team performed random allocation by drawing slots. The study process flowchart is presented in Figure 1.

### Participants

The study included participants who met the following criteria: (1) diagnosis of type 2 diabetes and receiving case management services for at least 3 months, (2) aged 20 years or older, (3) able to communicate in Mandarin or Taiwanese, and (4) possess a mobile phone or tablet with an internet connection. Patients with serious diseases such as general paralysis, mental disorders, and cognitive function abnormalities were excluded. Enrollment took place from July to September 2020, with the intervention period from September to December 2020, and the posttest completed in December 2020 and March 2021.

To detect a clinically significant effect, defined by the within or between-group interaction and follow-up time, on the outcome indicators, a sample size of 82 was calculated using repeated measures ANOVA between the factors based on an effect size of 0.25, as per Cohen guidelines [29]. This used an intercluster correlation coefficient of 0.5, a power of 0.8, and an α of .05. To account for a potential percentage dropout rate of 20%, the total sample size was increased to 92 participants. Ultimately, the study recruited 92 patients with type 2 diabetes.

### Procedure

We recruited patients with type 2 diabetes from various metabolic clinics throughout northern Taiwan. These clinics are integrated into Taiwan’s nationwide health insurance system to ensure all citizens have access to medical services. They are also part of the Diabetes Shared Care Program in Taiwan, which provides specialized care for individuals with metabolic disorders. In these clinics, patients receive comprehensive care from a multidisciplinary team comprising metabolic doctors, nurses, case managers, dieticians, and pharmacists. The services provided include the essential components of effective diabetes management such as regular laboratory tests, health examinations, and health education programs.

Before starting the study, our research team collaborated with the clinic management to establish a clear and mutually agreed-upon research process. To recruit participants, the clinics displayed posters providing information about the study. Research assistants were available to thoroughly explain the research procedures to interested individuals and obtain informed consent. Participants were asked to complete a pretest only after completing the explanation process and obtaining informed consent. This ensured a thorough and ethical approach to participant recruitment and data collection.

Next, after the preassessment, those willing participants were added with a case number. The research team used a computer program to draw slots, assigning the cases to the intervention or control group using a lottery system. The intervention group participated in a 3-month, 6-session eHealth care experiential learning program, while the control group received the usual care and an eHealth care manual. In Taiwan, the usual care refers to the standard treatment and follow-up in outpatient units. Typically, physicians schedule appointments every 3 months for patients with type 2 diabetes to conduct blood tests and prescribe medications. This care often includes health consultations by case managers or nutritional counseling. Both groups underwent posttesting twice: immediately after the 6-session course and 3 months later. Patients with chronic diseases usually have regular medical appointments every 3 months in Taiwan, as mandated by the national health insurance. As such, the control group completed their regular medical appointments during the same period.

### Intervention

The 6-session eHealth care experiential learning program (Multimedia Appendix 2), which focused on self-management of diabetes, was based on the eHL framework (eHLF) [30,31] and ELT cycle [20]. Over 3 years, our research team developed a program specifically designed for patients with type 2 diabetes and the challenges health care providers encounter in managing this condition. In the initial year, we began a qualitative study to gain in-depth insights into the needs and challenges associated with diabetes care. This study revealed several critical aspects for enhancing self-management among patients with diabetes. One of the pivotal insights from the study was the vital role of eHealth care for enhancing home care for patients, particularly those with chronic diseases such as diabetes, for which consistent health monitoring at home is essential. Patients’ 3-monthly clinical visits further highlighted the need for effective home-based health monitoring and guidance. These insights underscored their struggle to engage in meaningful health care discussions, often due to their unfamiliarity with eHealth technologies and a lack of knowledge on self-management practices.

In the program’s second year, we initially developed 8 sessions to address these identified gaps. However, after conducting a Delphi study and a pilot study to test the program’s feasibility and usability, we streamlined it to 6 sessions. This refinement process intended to provide a more effective and concentrated participant learning experience focused on the program content. The program’s overall development cost, including expenses for wearable devices and Bluetooth-enabled health monitors (eg, blood pressure monitors, glucose monitors, and scales), amounted to approximately US $66.

Therefore, the program followed the learning cycle, with the following activities and strategies, divided into 4 stages: concrete experience, reflective observation, abstract conceptualization, and active experimentation. First, the concrete experience stage introduced the importance of health care management and eHealth care for patients with diabetes. The program provided real-life examples and scenarios to help patients understand the significance of eHealth care in managing their condition.

Second, various methods were used in the reflective observation stage to encourage patients to reflect on their learning experiences. Videos were played to show the practical implementation of eHealth care. Group discussions were held to facilitate dialogue and share new insights among patients participating in the eHealth care program. Additionally, each session began with a review to help increase patients’ familiarity with eHealth care devices. This approach ensured a blend of visual learning and active participation, which enhanced the program’s overall effectiveness.

Third, the abstract conceptualization stage aimed to clarify the concepts of eHealth care using social media platforms such as LINE and Facebook and internet groups. Patients engaged in web-based discussions to deepen their understanding of eHealth care practices. Moreover, group discussions fostered dialogue among participants around their eHealth care practice experiences.

Finally, the active experimentation stage allowed patients to learn by actively engaging in related health situations. In this stage, they had the opportunity to choose suitable health technology software, hardware, and wearable devices for their specific diseases in a simulation environment.

Then, patients participated in group and individual competitions that involved using eHealth care tools effectively. They were assisted in setting practice and self-management goals and encouraged to use eHealth activities at home. Overall, this experiential learning program followed a systematic learning cycle that involved introducing concepts, facilitating reflection, deepening understanding, and encouraging active application and experimentation with eHealth care tools and techniques.

Our eHealth care experiential learning program comprised 6 biweekly sessions over 3 months, each lasting around 90 minutes. These sessions, conducted by the project investigator (YCC), combined theoretical knowledge with extensive hands-on training and at-home practice using various eHealth tools and applications commonly used in Taiwanese clinical settings. This included wearable devices such as Xiaomi, health care applications such as Health2Sync, LINE, My Health Bank, and blood pressure and glucose monitors. These hardware and software tools are designed to track various health records and statuses such as heart rates, sleep patterns, blood sugar levels, blood pressure, diet, and medication adherence. With Bluetooth connectivity, they seamlessly integrate into everyday health management and have been widely adopted in clinical settings due to their effectiveness and ease of use.

The six sessions were as follows:

1. “A new realm of health technology care”: Introduced smart health care functions, eHealth applications, and motivated patients to engage with eHealth technologies while reflecting on their health care experiences.
2. “Fighting against sugar”: Emphasized the importance of regular health monitoring (blood sugar, blood pressure, and diet) in diabetes care, focusing on self-management, demonstrating diabetes health care technology, and providing patients with practice tools.
3. “Trick of the trade for chronic kidney disease prevention”: Focused on diabetes complications, particularly chronic kidney disease, discussing self-management and relevant health care technologies that could be used for diabetic nephropathy.
4. “eHealth care by your side”: Used competitions and hands-on activities to foster patient engagement with health care technology. Patients were encouraged to operate the technology independently and discuss any challenges they faced. This approach not only motivated active participation but also facilitated learning from real-life experiences, enabling patients to better manage their health conditions using these technologies.
5. “My smart eHealth in daily care”: Focused on how health care technology can enhance patients’ lives. The participants were taught to correctly operate various eHealth components and integrate smart health care into their daily self-management. This approach emphasized practical use, encouraging patients to apply smart health care technologies to manage their health and benefit from their functionality in everyday life.
6. “My eHealth care practice journey”: Synthesized the key elements from the previous sessions. This final session focused on ensuring that patients could accurately operate smart health care applications, reinforcing their ability and willingness to engage in self-care management using health care technology. This comprehensive review and hands-on practice session aimed to solidify patients’ understanding and proficiency in applying these technologies for their ongoing health management.

Overall, these sessions aimed to provide patients with the knowledge and skills to effectively use eHealth tools for managing their health, enhancing their outcomes through technology-driven self-management. During each session, the project investigator (YCC) began by encouraging participants to ask questions and provide feedback on the previous session for 15 minutes. Following this, the project investigator introduced the current session’s content, and research assistants assisted participants in the practical operation of health care technology and tools for the remaining 15-20 minutes. After the session, participants could practice one-on-one and discuss their learning challenges with the research team. Additionally, between the 2 sessions, the research team used a social media platform to coach participants and provide feedback on any questions. Throughout the sessions, various teaching aids and equipment were used, including learning manuals, social media platforms, health applications, and wearable health devices. The participants in each session simultaneously experienced 4 ELT learning cycles. This approach enabled them to experience intelligent care and learn by practicing daily self-management using eHealth and engaging in the smart health care system.

### Data Collection

The data collection was blind to grouping. A single-blind method was applied. The clinics’ case managers collected the posttest data. The participants filled out the questionnaires independently during their medical appointments every 3 months. The case managers assisted participants if they required help with completing these questionnaires. All participants completed the questionnaires during admission (T0). The participants in the intervention group completed the posttest 1 (T1) questionnaires after completing the 3-month intervention program (T1) and again after a 3-month follow-up (T2) during their regular medical appointments. The participants in the control group completed the 2 posttest questionnaires during their regular medical appointments scheduled at 3 monthly intervals, 3 (T1) and 6 months (T2) after enrolling in this study.

### Measurement

This study used questionnaires to collect data, which included sociodemographic data, the eHealth Literacy Questionnaire (eHLQ), the Patient Health Engagement Scale, and the eHealth Care Use Scale (Multimedia Appendix 3 [13,32-34]).

#### Sociodemographic Data

Sociodemographic data included age, sex, education, economic status, number of comorbidities, perceived severity of illness, and health status.

#### eHLQ Instrument

The 35-item eHLQ is based on the eHLF, which refers to the ability to seek, find, understand, and appraise health information from electronic sources and apply the knowledge gained to address or solve a health problem. The eHLQ is typically structured to cover various dimensions of eHL, including technical skills, ability to understand health information, critical evaluation of web-based sources, and ability to actively engage with digital health technologies [13]. It comprises the following seven subscales: (1) using technology to process health information, (2) understanding the concept and language of health, (3) actively participating in technology service abilities, (4) feeling safe and self-controllable about personal health information, (5) motivation to participate in information technology services, (6) obtaining useful information technology services, and (7) information technology services that meet personal needs. Each item was scored on a 4-point scale (1=strongly disagree, 2=disagree, 3=agree, and 4=strongly agree). The higher the score, the better the patients’ eHL. The total score ranged from 35 to 140. The original scale’s reliability was 0.8, and it had good construct and discriminant validity [13]. The Chinese version of the eHLQ has been found to have a content validity of 0.97, and the Cronbach α for the entire scale was 0.98, with subscales ranging from 0.74 to 0.97 [32].

#### Patient Health Engagement Scale

Graffigna and Barello [33,35] developed the Patient Health Engagement Scale using the patient health engagement model. The scale investigates if patients are constantly engaging in their health [33,35]. It includes the following four positions along a continuum of engagement: (1) blackout, (2) arousal, (3) adhesion, and (4) eudaimonic project. The scale can conceptualize the psychological aspects of patient health engagement and includes 5 ordinal items. To avoid social desirability bias, 4 positions of raw patient health engagement were categorized into a 7-point scale: 1 and 2=blackout, 3 and 4=arousal, 5 and 6=adhesion, and 7=eudaimonic project. To acquire the final engagement position, the 4 positions of patient health engagement were arranged from lowest to highest. The median score corresponds to the third position, which represents the patient health engagement position. These 4 positions arise from conjoint cognitive (thinking), emotional (feeling), and conative (acting) enactment. The Cronbach α for this scale was 0.85, and its retest reliability was 0.95, indicating good reliability and validity [33]. The Chinese version of the scale translated by Zhang et al [34] had an internal consistency of 0.89 and a retest reliability between 0.52 and 0.79.

#### eHealth Care Use Scale

We used a self-developed scale to investigate various types of eHealth care use and monitoring items in daily disease management. The scale had high internal consistency (Cronbach α=0.92) and good content validity (content validity=0.95). The 4 types of eHealth care included were computer or internet systems, mobile apps, health monitoring systems, and wearable devices (eg, pedometers, smart bracelets, heart rate monitors, blood pressure monitors, blood glucose meters, and weight scales). The scoring method was as follows: 0=no use of any eHealth types, 1=use of 1 type, 2=use of 2 types, 3= use of 3 types, and 4=use of 4 types. Higher scores indicated a greater variety of eHealth types used.

Furthermore, the monitoring items encompassed blood pressure, blood sugar, weight, diet, sleep, heart rate, steps, and other health data. The scoring method for the monitoring items was as follows: 0=not monitoring any health data, 1=monitoring 1 type, 2=monitoring 2 types, 3=monitoring 3 types, 4=monitoring 4 types, 5=monitoring 5 types, 6=monitoring 6 types, and 7=monitoring 7 types. A higher score indicated a greater number of health data items being monitored. The total score reflected overall eHealth care use with a higher score indicating more comprehensive use.

### Statistical Analysis

The data were analyzed using SPSS (version 24.0; IBM Corp). Descriptive statistics were used to report continuous data using scores, means, and SDs. Categorical data were presented as numbers and percentages. Independent sample *t* tests (2-tailed) and chi-square tests (2-tailed) were used to compare the homogeneity of the pretest data between the 2 groups. To avoid overestimating the effect of the intervention while not ruling out loss to follow-up, intention-to-treat analysis was used.

The generalized estimating equation (GEE) was used to examine the difference in effectiveness, with statistical significance set at *P*<.05. This statistical analysis method can accommodate repeated responses from each participant. The autoregressive (1) model of the GEE using a linear regression model was used to estimate whether there was a significant difference in the improvement of the outcome indicators between the 2 groups over time. To reduce interference factors, sex and economic status—both of which showed significant differences in the initial test after randomization—were included as covariates in the corrected GEE model. This adjustment addresses the potential impact of sex and economic status imbalances and the interaction between time and group. Consequently, the reliability of our findings is enhanced despite the imbalance issue following randomization.

### Ethical Considerations

This study was approved by the institutional review board of the university with which the research team is affiliated (YM106120E-3). Before data collection, we provided the participants with an explanation of the research objectives, process, and questionnaire content, both verbally and in writing. Once participants completed a questionnaire at each time point—pretest, postintervention, and 3 months after the intervention—they were able to receive an NT $100 voucher (a currency exchange rate of NT $1=US $0.33 is applicable). We also assured them that their participation would be confidential and would affect neither their treatment rights nor their health care. Data were collected after the participants had signed the consent form. The participants were free to withdraw from the study at any point, even after providing consent.

## Results

### Participant Characteristics

A total of 86 participants completed the 2 posttests, including 41 (48%) in the intervention group and 45 (52%) in the control group. The total participant attrition rate was 7% (n=6). The rates of subject loss in the intervention and control groups were 11% (5/46) and 2% (1/46), respectively. The initial 92 participants were included in the final analysis (Table 1).

**Table 1:** Baseline comparison of the patients.

Variable		Total (N*=*92)	Intervention group (n*=*46)	Control group (n*=*46)	*t* test (*df*) or chi-square test (*df*)	
Age (years), mean (SD)		62.38 (13)	64.96 (10)	59.80 (15)	1.9 (90)^a^	
**Sex, n (%)**						8.0 (1)^b,c^
	Male	33 (36)	10 (22)	23 (50)		
	Female	59 (64)	36 (78)	23 (50)		
**Education, n (%)**						6.1 (4)^b^
	Elementary school and below	18 (20)	6 (13)	12 (26)		
	Junior high school	16 (17)	11 (24)	5 (11)		
	High or vocational school	32 (35)	14 (30)	18 (39)		
	College or university	21 (23)	13 (28)	8 (17)		
	Graduate school	5 (5)	2 (4)	3 (7)		
**Economic status (NT $^d^), n (%)**						9.7 (4)^b,c^
	<10,000	29 (32)	16 (35)	13 (28)		
	10,001-20,000	24 (26)	6 (13)	18 (39)		
	>20,001-30,000	19 (21)	13 (28)	6 (13)		
	>30,001-40,000	15 (16)	9 (20)	6 (13)		
	>40,001	5 (5)	2 (4)	3 (7)		
**Number of comorbidities,** **mean (SD)**		1.98 (1)	1.83 (1)	2.13 (1)	–1.4 (90)^a^	
	1 item, n (%)	38 (41)	20 (44)	18 (39)		
	≧2 items, n (%)	54 (59)	26 (57)	28 (61)		
**Perceived severity of illness, n (%)**						0.8 (2)^b^
	Not serious	28 (30)	12 (26)	16 (35)		
	Normal	62 (67)	33 (72)	29 (63)		
	Serious	2 (2)	1 (2)	1 (2)		
**Health status, n (%)**						4.3 (2)^b^
	Bad	18 (20)	7 (15)	11 (24)		
	Normal	54 (59)	26 (57)	28 (61)		
	Good	20 (22)	13 (28)	7 (15)		
eHealth literacy, mean (SD)		93.82 (19)	90.96 (19)	96.67 (18)	–1.5 (90)^a^	
Patient health engagement, mean (SD)		2.92 (1)	2.91 (1)	2.96 (1)	–0.5 (90)^a^	
eHealth care use, mean (SD)		2.27 (2)	2.17 (2)	2.37 (2)	–0.4 (90)^a^	

^a^Independent sample *t* test (2-tailed).

^b^Chi-square test (2-tailed).

^c^The difference between the 2 groups at a significance level of .01 (2-tailed).

^d^A currency exchange rate of NT $1=US $0.33 is applicable.

The mean age of the 92 participants was 62.38 (SD 12.9) years, and 59 (64%) were female participants, while 33 (36%) were male participants. Regarding education level, the highest proportion had a high school or higher vocational education, accounting for 32 (35%) participants. The intervention group consisted mostly of female participants, whereas the control group had an equal distribution of male and female participants. There was a significant difference in sex between the 2 groups (*χ*^2^_1_=8.0, *P*=.005). Regarding economic status, the intervention group had more participants with an income of NT $20,000-30,000, while the control group had more participants earning NT $10,000-20,000. There was a significant difference in economic status between the 2 groups (*χ*^2^_4_=9.7, *P*=.046). In terms of comorbidities, 54 (59%) individuals had at least 2 types of chronic diseases. In contrast, 38 (41%) individuals had 1 type. Moreover, 62 (67%) participants indicated a general level of subjective disease severity. Similarly, regarding self-rated health status, 54 (59%) participants considered their health status general (Table 1).

There were no significant differences in most characteristics between the 2 groups, except for sex and economic status. Furthermore, the baseline assessment of the outcome indicators, including eHL, patient health engagement, and eHealth care use, showed no significant differences.

### Effectiveness of the eHealth Care Experiential Learning Program

**Figure 2:**
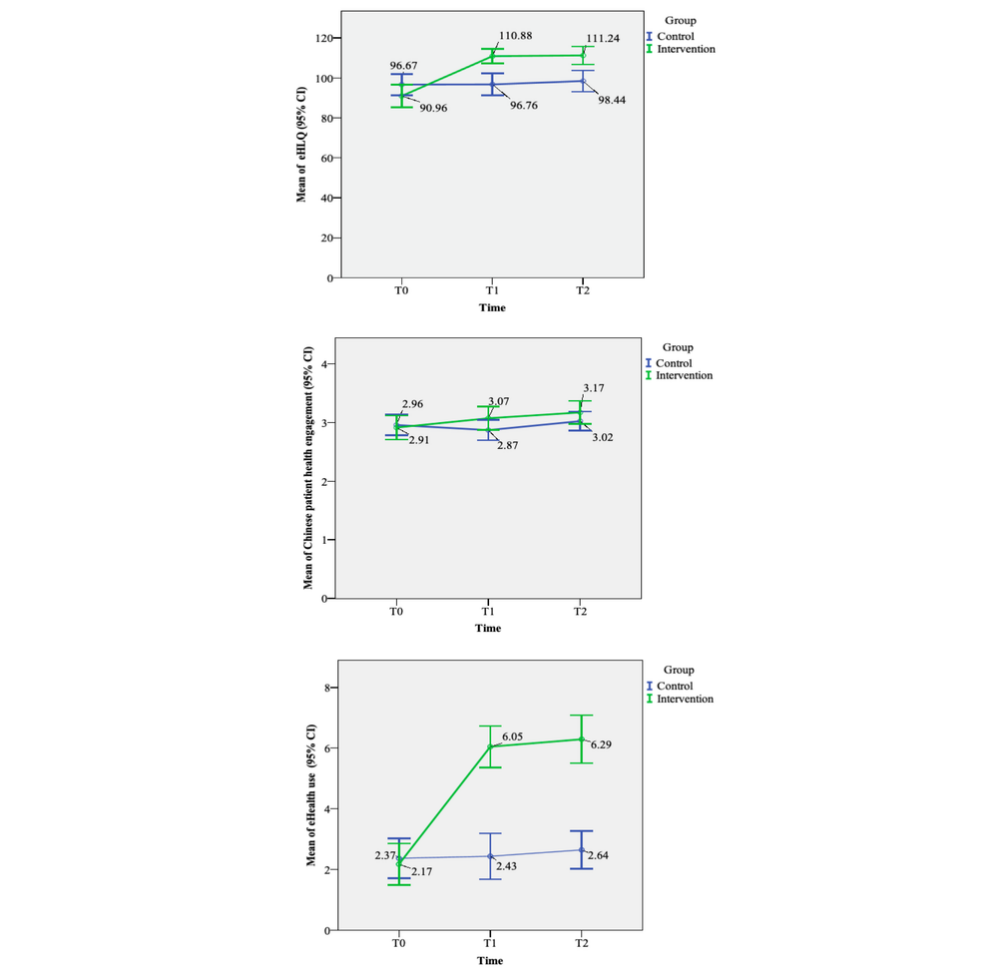
Effects of the eHealth care experiential learning program on eHealth literacy, patient health engagement, and eHealth care use among patients with type 2 diabetes. eHLQ: eHealth Literacy Questionnaire.

The results revealed significant differences in the patients’ eHL, patient health engagement, and eHealth use after participating in the eHealth care experiential learning program (Table 2, Multimedia Appendix 4, and Figure 2). Moreover, there were also significant differences in the eHL subscales (Multimedia Appendix 5).

**Table 2:** Mean changes in eHealth literacy, patient engagement, and eHealth care use over time (N=92).

				β (SE)		*P* value	
**eHealth literacy**							
	Intercept			91.12 (4.06)	<.001^a^		
	Group (intervention vs control)			–6.11 (3.58)	.09		
	**Time**						
		T2 vs T0	2.11 (2.03)				.30
		T1 vs T0	.09 (1.39)				.95
	**Sex**						
		Female vs male	–1.19 (3.08)				.70
	**Economic status (NT $^b^)**						
		>40,001 versus <10,000	13.95 (4.63)				.003
		>30,001-40,000 versus <10,000	8.41 (4.08)				.04
		>20,001-30,000 versus <10,000	13.55 (3.78)				<.001^a^
		10,001-20,000 versus <10,000	6.08 (4.21)				.15
	Group*time						
		Intervention*T2 versus control*T2	18.19 (3.82)				<.001^a^
		Intervention*T1 versus control*T1	19.94 (3.52)				<.001^a^
**Patient health engagement**							
	Intercept			3.05 (0.11)	<.001^a^		
	Group (intervention vs control)			–.07 (0.14)	.59		
	**Time**						
		T2 versus T0	.05 (0.08)				.52
		T1 versus T0	–.09 (0.08)				.28
	**Sex**						
		Female versus male	–.12 (0.12)				.32
	**Economic status (NT $^b^)**						
		>40,001 versus <10,000	–.43 (0.28)				.12
		>30,001-40,000 versus <10,000	–.06 (0.17)				.73
		>20,001-30,000 versus <10,000	.15 (0.13)				.25
		10,001-20,000 versus <10,000	–.05 (0.13)				.70
	**Group×time**						
		Intervention**×**T2 versus control**×**T2	.24 (0.14)				.07
		Intervention**×**T1 versus control**×**T1	.28 (0.13)				.04^c^
**eHealth care** **use**							
	Intercept			1.54 (0.47)	.001		
	Group (intervention vs control)			–.21 (0.48)	.66		
	**Time**						
		T2 versus T0	.35 (0.30)				.23
		T1 versus T0	.07 (0.25)				.79
	**Sex**						
		Female versus male	.30 (0.44)				.49
	**Economic status**						
		>40,001 versus <10,000	2.16 (0.60)				<.001^a^
		>30,001-40,000 versus <10,000	.70 (0.57)				.22
		>20,001-30,000 versus <10,000	.97 (0.55)				.08
		10,001-20,000 versus <10,000	.82 (0.55)				.14
	**Group** **×** **time**						
		Intervention**×**T2 versus control**×**T2	3.87 (0.49)				<.001^a^
		Intervention**×**T1 versus control**×**T1	3.96 (0.42)				<.001^a^

^a^The difference between the 2 groups at a significance level of .001 (2-tailed).

^b^A currency exchange rate of NT $1=US $0.33 is applicable.

^c^The difference between the 2 groups at a significance level of .05 (2-tailed).

The intervention group showed a significantly higher increase in eHL scores than the control group at both posttest time points. Specifically, the improvement in the intervention group was greater than that in the control group (β=19.94, SE 3.52; *P*<.001 and β=18.19, SE 3.82; *P*<.001) at T1 and T2. These findings indicate that the intervention positively improved eHL, which persisted for up to 3 months afterward.

Similarly, patient health engagement increased following the intervention, with a higher mean score change in the intervention group than in the control group. Specifically, the improvement in the intervention group was significantly higher than in the control group (β=.28, SE 0.13; *P*=.03) at the initial posttest (T1). However, at T2 (3 months after the intervention), there was no significant difference between the 2 groups in the extent of the change (β=.25, SE 0.14; *P*=.06). The effect of the learning program on the patients’ eHealth use was also examined. We compared the test scores of the intervention group at the 2 posttest time points and found that the mean score increase in the intervention group was higher than that in the control group. Specifically, the improvement in the intervention group was significantly greater than in the control group at T1 and T2 (β=3.96, SE 0.42; *P*<.001 and β=3.87, SE 0.49; *P*<.001).

## Discussion

### Principal Findings

This study examined the impact of an experiential learning program focused on eHealth care among individuals with type 2 diabetes. We found significant improvements in the intervention group’s eHL, patient health engagement, and eHealth use compared with the control group following a series of educational sessions. The experiential learning program immersed participants in daily care activities and provided real-life experiences, resulting in a low attrition rate and positive acceptance of health care technology. These findings indicate that the program effectively enhanced participants’ skills, attitudes, and behaviors. Enhancing eHL is crucial in the rapidly evolving eHealth era, in which an increasing array of eHealth devices is becoming available. When equipped with adequate eHL, patients with chronic diseases are better positioned to engage in self-management and address health inequities [36].

While many previous studies have conducted eHealth interventions, their findings on the effectiveness of eHealth care vary [37]. This theory-based eHealth intervention focuses on practical, experiential learning and the learning cycle, thus bridging this gap in the research because of its broad generalizability. After participants joined the program, we also found that increasing eHL is crucial to improving their health-related behavior. Patients became more actively involved in self-management when using the eHealth system. Consequently, this program demonstrates the potential for future application in clinical care, enabling patients to actively engage in a smart eHealth care system, implement self-management skills, and maintain disease control to improve quality of life. Moreover, validating our program with a clinical population of patients with diabetes underscores its clinical relevance and applicability, thereby demonstrating its value in real-world health care settings. The successful implementation and positive outcomes in this context indicate that the program can also be used to manage other chronic conditions.

The study found that participation in experiential learning courses effectively improved individuals’ eHL, enabling them to use technology for daily self-management tasks. The course curriculum was based on the eHLF, encompassing all stages of the learning process. By engaging in experiential learning activities, participants applied learning cycles and integrated teaching strategies mirroring real-world situations in chronic disease management. This approach aligns with Kolb’s theory, emphasizing learning through everyday life experiences, where knowledge is generated and transformed through practical engagement [19,20]. Learners fully engage in learning by incorporating active participation and hands-on experiences, fostering interest, encouraging questioning, and promoting reflective practices [20]. Our program comprised activities that closely resembled real-life situations, adhering to the principles of the eHLF to promote active participation among the learners. For example, during the concrete experience stage, we introduced eHealth care for the self-management of chronic diseases through lectures and videos to enhance patients’ understanding of eHealth care. This approach enhances their interest in digital services, prompting them to take a more active role in managing their health, as indicated by the increased patient health engagement shown in Figure 2. Moreover, in the active experimentation stage, we assisted patients in setting personal goals and facilitated hands-on learning by using various eHealth care devices. These activities provided them with access to appropriate digital services tailored to their health care needs and were designed to meet the unique challenges and requirements of the learners. As depicted in Figure 2, the intervention group recorded higher scores in eHealth care use than the control group. Through these experiential learning opportunities, participants gain knowledge and insights that evolve into personal skills they can integrate into their daily lives. This process empowers them to make informed decisions about their health and well-being, ultimately enhancing their eHL. By engaging in experiential learning courses, participants develop the necessary skills and confidence in the digital health domain [22,38].

Participants demonstrated enhanced overall eHL and significant progress in all 7 subscales. Specifically, notable improvements were observed in subscale 1 (using technology to process health information) and subscale 2 (understanding health concepts and language). To improve the ability to process information, the program effectively incorporated sessions that elucidated the importance of disease management, demonstrated appropriate health care technology applications, and introduced the concept of smart health care. This comprehensive learning program enabled participants to internalize these concepts and cultivate their abilities while actively engaging them in health care. Previous research has shown that explaining the principles that necessitate improvement not only enhances knowledge and skills but also diminishes barriers to implementation [20,26]. We also observed evident improvements in subscales 3, 5, 6, and 7.

To enhance their understanding of eHealth care, we introduced the patients in this study to various eHealth applications such as smart health care applications, wearable devices, and social media through case studies, lectures, and videos. We also used visual aids, questions, and case studies to enhance their knowledge about health care technology. Thus, patients could select suitable health care technologies that meet their individual care needs. Previous studies have indicated that higher health awareness makes it easier for individuals to comprehend health-related information and actively seek to improve their self-management abilities [6,39]. People can integrate knowledge through practical experience, case studies, and handouts [22,24].

In addition, the issue of information security often affects willingness to use eHealth technologies; however, subscale 4 showed clear improvements in this study. Each session provided information about health-related security, ways to preserve health information, and strategies for fraud prevention. Moreover, we encouraged patients to ask questions and express their concerns, which were clarified by the research team. Patients were informed about security measures and policies and how health care professionals use them. As certain software and tools can determine authenticity, patients’ trust in technological information can be promoted through our intervention. Previous studies have suggested that asking and answering questions [40], assisting in the operation [26], and clarifying concerns [41] can help build people’s trust in their abilities.

We also observed an improvement in patient health engagement. Although there was no significant difference in patient health engagement after 3 months, the level of engagement reached the arousal stage. Patient health engagement is a continuous, dynamic psychological process through which patients’ cognition, emotions, and behaviors interact. This indicates that participants gradually accepted their illness and actively started to engage in disease management [35]. Previous research has highlighted the lack of knowledge on self-management of diseases and how to effectively use tools for disease management, leading to low engagement [6,42]. In this study, implementing the eHLF helped systematically organize participants’ care needs and connect them to relevant eHealth applications. This enabled patients to understand, take action, and gradually internalize these practices while passing through the learning cycle. Faiola et al [42] also emphasized providing systematic mobile-based health frameworks based on patients’ needs, empowering lifestyle changes, and promoting sustainable healthy behaviors.

Additionally, patient health engagement was measured on a 5-point scale, making it difficult to observe significant changes. Nevertheless, Figure 2 demonstrates the rise in patient health engagement, which can be attributed to patients learning to use health care technology daily and setting goals to track their health status. Through this program, we helped patients accumulate concrete, real-life self-care experiences, transformed abstract care concepts into usable knowledge, and assisted in operating health technology applications. Using their life experiences in case studies and addressing their questions reduced anxiety about technology use, promoted patient identification, and facilitated learning and engagement [6,43]. Furthermore, patients also participated in group discussions, which previous studies have shown to increase their sense of reality and engagement with eHealth care services [22,23]. This further supported patients’ engagement by fostering a sense of community and shared experiences. As a result, patients are actively engaged with health care technology in their daily lives.

Finally, the intervention significantly improved participants’ eHealth use. Our experiential learning program encouraged patients to participate in 6 sessions of the experience cycle. During these sessions, we demonstrated the operation of eHealth care devices and assisted each patient in selecting suitable devices according to their health conditions and abilities. Additionally, we provided simulation scenarios related to eHealth care, allowing patients to gradually familiarize themselves with operating these health care devices. Patients were encouraged to ask questions, and immediate answers were provided. Once taught, patients could use health technology for self-management independently. Educating individuals based on ELT can enhance their learning and skills [4,22,26]. Moreover, patients who engage in hands-on practice and receive information from simulation scenarios experience reduced fear and increased acceptance of new things [38]. Furthermore, we used LINE, a communication app, to remotely accompany patients in their daily lives. A web-based patient support group was established that allowed patients to ask questions at any time and receive emotional support. We also addressed patients’ questions individually whenever they encountered difficulties in using eHealth care. As a result, patients gained problem-solving skills and overcame any fear or hesitation in using health technology. These findings align with previous studies showing that when patients recognize the need to use such devices, they become more willing to share their needs and engage in discussions [9,43,44]. Additionally, when patients truly experience the benefits of using health technology, they integrate it as a helpful and effective part of their lives [45,46]. Therefore, our program has the potential to sustain patients’ eHealth use.

### Limitations

This study has several limitations. The 3-month follow-up period may not fully capture the long-term effectiveness of the intervention. Recognizing the importance of longer follow-ups for assessing the sustainability of health behaviors and disease management outcomes, future research should consider extended follow-up periods to more accurately assess health behavior and health outcome changes. Moreover, several strategies can be implemented to sustain the long-term effects of eHealth care experiential learning program. Empowering patients to participate actively in their health management is crucial. Continuous support and counseling through regular refresher sessions can help reinforce learning and maintain patient engagement. Therefore, integrating the program into routine clinical practice is essential, involving health care providers in the ongoing monitoring and supporting patients’ eHealth activities to maintain the program’s benefits. Providing peer support and fostering a shared responsibility for health management can also contribute to sustaining the program’s impact. The extended follow-up includes collecting data on motivation (empowerment), health behaviors (such as patient health engagement, self-management, and eHealth use), and disease control indicators such as blood sugar levels, hemoglobin A_1c_ %, and lipid profiles. We will share these long-term findings in future publications. Additionally, a sex disparity was observed, with female participants being more active in health-related activities than male participants, partly due to the higher employment rates among male participants limiting their availability. Future studies should explore strategies to increase male’s participation to improve external validity. Furthermore, this study’s reliance on quantitative data may overlook insights that qualitative research can provide. Incorporating qualitative approaches in future research could yield a deeper understanding of user perspectives and the effectiveness of the ELT-based intervention. Addressing these limitations would enhance our understanding of the intervention’s long-term impact and its applicability to a broader population of patients with chronic illnesses.

### Conclusions

This study provided compelling evidence of theory-based interventions in eHealth care, displaying their effectiveness in improving eHL, patient health engagement, and eHealth use. By applying ELT, patients with type 2 diabetes demonstrated notable enhancements in their abilities and skills to use eHealth care technology. This approach clarifies the generalizability of the intervention and its components and contributes significantly to bridging the research gap in understanding the impact of eHL on patient behavior. A key finding from this study, after participants engaged with the program, was the crucial role of increasing eHL in improving patients’ health-related behaviors. The study further underscored the significance of the eHLF as a fundamental knowledge base, acquired through immersive, real-world, self-management tasks using eHealth care tools within the learning cycle by demonstrating the tangible benefits of a theory-driven eHealth intervention in a clinical setting. The interventions designed to mimic patients’ daily life situations closely facilitated the optimal learning outcomes and consequent changes in their health behavior. As such, integrating patient-centered care becomes imperative in the health care system to actively involve patients in eHealth care programs and promote improved disease management in the future.

## Appendix

Multimedia Appendix 1 CONSORT (Consolidated Standards of Reporting Trials) checklist.

Multimedia Appendix 2 Content of the eHealth care experiential learning program.

Multimedia Appendix 3 The study questionnaire.

Multimedia Appendix 4 Mean changes in the measures of eHealth literacy, patient health engagement, and eHealth use over time (N=92).

Multimedia Appendix 5 Mean changes in the 7 eHealth literacy subscales over time (N=92).

## Abbreviations

CONSORT: Consolidated Standards of Reporting Trials
eHL: eHealth literacy
eHLF: eHealth literacy framework
eHLQ: eHealth Literacy Questionnaire
ELT: experiential learning theory
GEE: generalized estimating equation
