# Supplementary material for: Short-Term Effects of an eHealth Care Experiential Learning Program Among Patients With Type 2 Diabetes: Randomized Controlled Trial
Source: J Med Internet Res. 2024 Aug 16;26:e53509. doi: 10.2196/53509 (PMC11364949; doi:10.2196/53509)
Supplement: Multimedia Appendix 2 [file jmir_v26i1e53509_app2.docx]

| Session | eHealth Literacy Framework | Teaching Strategies and Activities |
| --- | --- | --- |
| 1. A new realm of health technology care | 1. Ability to process information 2. Understanding health concepts and language 3. Motivated to engage with digital services | 1. Concrete Experience  - Introducing the concept of smart care and current eHealth applications, including computer networks, smart care applications, and wearable devices, through lectures.  1. Reflective Observation  - Understanding eHealth as a healthcare service through videos.  1. Abstract Conceptualization  - Dialogizing about one’s experience of using eHealthcare through group discussions.  1. Active Experimentation  - Self-experiencing eHealth devices and tools such as blue Bluetooth blood glucose, body weight, and blood pressure machine and app. |
| 1. Fighting against sugar | 1. Ability to process information 2. Understanding health concepts and language 3. Motivated to engage with digital services 4. Access to digital services that work | 1. Concrete Experience  - Introducing the importance of diabetes self-management, self-monitoring items, and diabetes home care through lectures and discussions. - Demonstrating smart care and the current eHealth application in diabetes care.  1. Reflective Observation  - Reflecting on one’s self-management behaviors and how to improve by using eHealth care.  1. Abstract Conceptualization  - Dialogizing about one’s experience of eHealth care practice through group discussions.  1. Active Experimentation  - Learning by doing through practicing using blood sugar, blood pressure, and body fat meter machines. |
| Session | eHealth Literacy Framework | Teaching Strategies and Activities |
| 1. Trick of the trade for chronic kidney disease prevention | 1. Ability to process information 2. Understanding health concepts and language 3. Motivated to engage with digital services 4. Access to digital services that work | 1. Concrete Experience  - Introducing the risk factors, symptoms, and health data of chronic kidney disease and how to prevent the progression of diabetes to chronic kidney disease through lectures and discussions. - Demonstrating smart care and current eHealth applications in chronic kidney disease care.  1. Reflective Observation  - Providing simulation scenarios of eHealth care for patients to initiate discussions.  1. Abstract Conceptualization  - Dialogizing about one’s experience of eHealth care practice through group discussions. - Sharing personal reflections on daily eHealth care in diabetes care practice.  1. Active Experimentation  - Teaching how to integrate home self-care with smart care. - Teaching how to use eHealth care applications, such as recording diaries, and search for health information. |
| 1. eHealth care by your side | 1. Ability to actively engage with digital services 2. Feeling safe about access to one’s personal health information 3. Motivated to engage with digital services 4. Access to digital services that work | 1. Concrete Experience  - Introducing current evidence-based research about the benefits of using smart health care for self-management through lectures.  1. Reflective Observation  - Encouraging patients to share their personal experiences of acquisition and challenges in using eHealth care.  1. Abstract Conceptualization  - Dialogizing about one’s experience of eHealth care practice through group discussions. - Sharing personal reflections from daily practice.  1. Active Experimentation  - Learning by doing by using eHealth care devices in competitive activities. - Learning by doing through setting personal health goals. |
| Session | eHealth Literacy Framework | Teaching Strategies and Activities |
| 1. My smart eHealth in daily care | 1. Understanding health concepts and language 2. Ability to actively engage with digital services 3. Feeling safe about access to one’s personal health information 4. Motivated to engage with digital services 5. Access to digital services that work 6. Digital services that meet individual needs | 1. Concrete Experience  - Guiding patients to choose suitable eHealth resources based on their health condition care needs through real-world care situations.  1. Reflective Observation  - Assisting patients in identifying which aspects of their lifestyle can be changed and in setting personal health goals. - Guiding patients to exchange their eHealth practice experiences in daily care tasks.  1. Abstract Conceptualization  - Discussing the plans and rules of group and individual competitions of using eHealth care tools. - Guiding patients to exchange their eHealth practice experiences in daily care tasks.  1. Active Experimentation  - Learning by doing by using eHealth care devices in competitive activities. - Learning by doing through setting personal health goals. - Practicing integrating home self-care activities with smart care. |
| 1. My eHealth care practice journey | 1. Ability to process information 2. Understanding health concepts and language 3. Ability to actively engage with digital services 4. Feeling safe about access to one’s personal health information 5. Motivated to engage with digital services 6. Access to digital services that work 7. Digital services that meet individual needs | 1. Concrete Experience  - Reviewing the advantages of smart care in chronic care through lectures and discussions.  1. Reflective Observation  - Reflecting on the changes in self-management through using smart care.  1. Abstract Conceptualization  - Guiding patients to use suitable eHealth resources based on their health conditions and care needs. - Reviewing the impact of the eHealth care learning process.  1. Active Experimentation  - Integrating home self-care with smart care by using eHealth care devices through learning by doing. - Celebrating the completion of competitive activities. - Presenting awards to groups and individuals for meeting their goals of using eHealth care. |
