# Supplementary material for: Short-Term Effects of an eHealth Care Experiential Learning Program Among Patients With Type 2 Diabetes: Randomized Controlled Trial
Source: J Med Internet Res. 2024 Aug 16;26:e53509. doi: 10.2196/53509 (PMC11364949; doi:10.2196/53509)
Supplement: Multimedia Appendix 3 [file jmir_v26i1e53509_app3.docx]

**一、個人資本資料**

1. 性別：□男 □女
2. 歲數：＿＿＿＿歲
3. 最高學歷：□小學及以下 □初中、國中 □高中、高職 □專科/大學以上
4. 您的慢性疾病有哪些：□糖尿病 □高血壓 □心血管疾病 □痛風 □高血脂 □腎臟疾病
   □其他__________
5. 自覺目前疾病嚴重程度：□非常不嚴重 □有點嚴重 □普通 □很嚴重 □非常嚴重
6. 自我健康狀況：□很差 □差 □普通一般 □好 □很好
7. 您過去一年的個人每月可自由運用的平均額度約多少元？□無收入 □1萬元以下 □1-2萬元
    □>2-3萬元 □>3-4萬元 □>4-5萬元 □>5萬元

**二、資訊科技化健康識能量表 (eHLQ)^a^ [13, 32]**

資訊科技（例如：手機、網路、雲端等）已融入我們生活當中，提升了大眾的健康與生活品質。許多科技化健康照護，例如：手機APP、網路掛號等，民眾是否獲得資訊科技帶來的便利以提升健康，請您針對以下每個陳述，勾選一個您認為同意程度的選項：

| 1. 運用科技來處理健康資訊 | |
| --- | --- |
|  | 我使用科技找…… |
|  | 我經常使用科技…… |
|  | 科技幫助我決定怎樣…… |
|  | 我使用科技分享……. |
|  | 我使用科技來…… |
| 1. 了解健康概念和語言 | |
|  | 我具備的知識有助於…… |
|  | 關於我的健康我有足夠….. |
|  | 我了解關於我自己……. |
|  | 整體而言，我了解我的…… |
|  | 我使用身體的測量….. |
| 1. 主動參與數位服務的能力 | |
|  | 我知道如何使用科技取得…….. |
|  | 我知道如何受惠於科技…… |
|  | 我能夠將資料輸入….. |
|  | 我很快地學會如何….. |
|  | 我很容易學會….. |
| 1. 感到安全和控制 | |
|  | 我確定只有應該使用的人……. |
|  | 我的電子健康照護…… |
|  | 我清楚地了解健康照護人員…… |
|  | 我確定只有被授權的人…… |
|  | 我有信心健康照護人員….. |
| 1. 被激發參與數位服務 | |
|  | 科技讓我覺得能主動……. |
|  | 我發覺科技可幫助…. |
|  | 當我使用科技時….. |
|  | 科技促進我….. |
|  | 我發覺科技有助…… |
| 1. 取得的數位服務是可用的 | |
|  | 需要關於我健康資訊的人…… |
|  | 我的健康照護人員提供的服務…….. |
|  | 不論我在任何地方…… |
|  | 所有我使用的健康科技…… |
|  | 我的大部分醫療照護人員…… |
|  | 我接觸過的健康科技…… |
| 1. 數位服務符合個人需求 | |
|  | 我發覺健康科技服務….. |
|  | 我發覺健康科技….. |
|  | 我發覺健康科技服務…… |
|  | 健康科技服務提供…… |

^a^Items are truncated. The full list of items is available with the authors.

**三、健康科技使用經驗與情況**

您曾使用過哪些方式監測及記錄生理數值、睡眠及飲食：

| 使用方式 | 監測項目（複選） |
| --- | --- |
| □紙本 | □未使用過 □血壓 □血糖 □體重 □飲食 □睡眠  □其他___________________ |
| □電腦或網路系統 | □未使用過 □血壓 □血糖 □體重 □飲食 □睡眠  □其他___________________ |
| □手機健康相關App | □未使用過 □血壓 □血糖 □體重 □飲食 □睡眠  □其他___________________ |
| □健康監測系統或穿戴裝置  （如計步器、健康智慧手環、心率監測、血壓計、血糖計或體重計等） | □未使用過 □血壓 □血糖 □體重 □飲食 □睡眠  □其他___________________ |
| □其他_____________ | □未使用過 □血壓 □血糖 □體重 □飲食 □睡眠  □其他___________________ |

**四、病患健康參與量表^a^ [33,34]**

以下是有關人們談論**自己健康狀況**時的個人體驗，請依據您個人的實際狀況，在每個選項中**圈出最能準確表達您目前感受狀態的分數**。

| **當我想起自己的疾病（或健康狀況）時：** | | | | |
| --- | --- | --- | --- | --- |
| **1** | 我感覺腦中…… | 我很…… | 我漸漸有…… | 我感到…… |
|  | **1 2 3 4 5 6 7** | | | |
| **2** | 我感到…… | 我很…… | 我了解自己的…… | 我感到很…… |
|  | **1 2 3 4 5 6 7** | | | |
| **3** | 一旦想起我的疾病，我感到…… | 每當出現新的症狀時，我就會…… | 我已經習慣了…… | 儘管我生病了，  我覺得…… |
|  | **1 2 3 4 5 6 7** | | | |
| **4** | 因為我自身的疾病我感到…… | 當我嘗試管理自己的疾病時，我感到…… | 我已經適應了…… | 我通常都對自己的未來和健康…… |
|  | **1 2 3 4 5 6 7** | | | |
| **5** | 我感覺自己完全…… | 當新的症狀出現時，我很…… | 我已經接受了…… | 儘管我生病了，  我也能…… |
|  | **1 2 3 4 5 6 7** | | | |

^a^Items are truncated. The full list of items is available with the authors.
