# Supplementary material for: Short-Term Effects of an eHealth Care Experiential Learning Program Among Patients With Type 2 Diabetes: Randomized Controlled Trial
Source: J Med Internet Res. 2024 Aug 16;26:e53509. doi: 10.2196/53509 (PMC11364949; doi:10.2196/53509)
Supplement: Multimedia Appendix 4 [file jmir_v26i1e53509_app4.docx]

| **Variable** | **Intervention group (*n* = 46)** | **Control group (*n* = 46)** | **β** | **SE^c^** | ***P value*** |
| --- | --- | --- | --- | --- | --- |
|  | **Mean (SD)** | **Mean (SD)** |  |  |  |
| **eHealth literacy** |  |  |  |  |  |
| T0 | 90.96 (18.81) | 96.67 (18.77) |  |  |  |
| T1^d^ | 110.88 (11.97) | 96.76 (18.46) | 0.09 | 1.39 | <.001 |
| T2^e^ | 111.24 (14.24) | 98.44 (14.24) | 2.11 | 2.03 | <.001 |
| Group^f^ |  |  | -6.11 | 3.58 | 0.09 |
| Group*×* T1^g^ |  |  | 19.94 | 3.52 | <.001^b^ |
| Group*×* T2^g^ |  |  | 18.19 | 3.82 | <.001^b^ |
| **Patient health engagement** |  |  |  |  |  |
| T0 | 2.91 (0.70) | 2.96 (0.60) |  |  |  |
| T1^d^ | 3.07 (0.64) | 2.87 (0.58) | -0.09 | 0.08 | 0.28 |
| T2^e^ | 3.17 (0.63) | 3.02 (0.54) | 0.05 | 0.08 | 0.52 |
| Group^f^ |  |  | -0.07 | 0.14 | 0.59 |
| Group*×* T1^g^ |  |  | 0.28 | 0.13 | 0.04^a^ |
| Group*×* T2^g^ |  |  | 0.24 | 0.14 | 0.07 |
| **eHealth care use** |  |  |  |  |  |
| T0 | 2.17 (2.31) | 2.37 (2.22) |  |  |  |
| T1^d^ | 6.05 (2.20) | 2.43 (2.55) | 0.07 | 0.25 | 0.79 |
| T2^e^ | 6.29 (2.50) | 2.64 (2.09) | 0.35 | 0.30 | 0.23 |
| Group^f^ |  |  | -0.21 | 0.48 | 0.66 |
| Group*×* T1^g^ |  |  | 3.96 | 0.42 | <.001^b^ |
| Group*×* T2^g^ |  |  | 3.87 | 0.49 | <.001^b^ |

Results were obtained using the generalized estimating equation (GEE) method.

^a^The difference between the two groups at a significance level of .05 (two-tailed)

^b^The difference between the two groups at a significance level of .001 (two-tailed)

^c^SE: Standard Error

^d^GEE results are for the change from baseline to 3 months. Reference: baseline

^e^GEE results are for the change from baseline to 6 months. Reference: baseline

^f^Reference: intervention group

^g^Reference: intervention group × baseline
