# Supplementary material for: Short-Term Effects of an eHealth Care Experiential Learning Program Among Patients With Type 2 Diabetes: Randomized Controlled Trial
Source: J Med Internet Res. 2024 Aug 16;26:e53509. doi: 10.2196/53509 (PMC11364949; doi:10.2196/53509)
Supplement: Multimedia Appendix 5 [file jmir_v26i1e53509_app5.docx]

|  | β (SE)^d^ | *P* value |
| --- | --- | --- |
| **1. Using technology to process health information** |  |  |
| **Intercept** | 12.12 (0.74) | <.001^c^ |
| **Group*time^a^** |  |  |
| Intervention*T2 versus control*T2 | 2.08 (0.67) | .002^b^ |
| Intervention*T1 versus control*T1 | 2.90 (0.59) | <.001^c^ |
| **2. Understanding health concepts and language** |  |  |
| **Intercept** | 13.61 (0.54) | <.001^c^ |
| **Group*time^a^** |  |  |
| Intervention*T2 versus control*T2 | 2.47 (0.50) | <.001^c^ |
| Intervention*T1 versus control*T1 | 2.38 (0.51) | <.001^c^ |
| **3.** **Ability to actively engage with digital services** |  |  |
| **Intercept** | 12.30 (0.78) | <.001^c^ |
| **Group*time^a^** |  |  |
| Intervention*T2 versus control*T2 | 2.40 (0.64) | <.001^c^ |
| Intervention*T1 versus control*T1 | 2.84 (0.58) | <.001^c^ |
| **4.** **Feeling safe and in control** |  |  |
| **Intercept** | 14.78 (0.56) | <.001^c^ |
| **Group*time^a^** |  |  |
| Intervention*T2 versus control*T2 | 2.53 (0.55) | <.001^c^ |
| Intervention*T1 versus control*T1 | 2.30 (0.53) | <.001^c^ |
| **5.** M**otivated to engage with digital services** |  |  |
| **Intercept** | 12.71 (0.79) | <.001^c^ |
| **Group*time^a^** |  |  |
| Intervention*T2 versus control*T2 | 2.77 (0.72) | <.001^d^ |
| Intervention*T1 versus control*T1 | 3.43 (0.65) | <.001^d^ |
| **6.** **Access to digital services that work** |  |  |
| **Intercept** | 15.55 (0.75) | <.001^c^ |
| **Group*time^a^** |  |  |
| Intervention*T2 versus control*T2 | 3.70 (0.68) | <.001^c^ |
| Intervention*T1 versus control*T1 | 3.50 (0.62) | <.001^c^ |
| **7. Digital services that suit individual needs** |  |  |
| **Intercept** | 10.03 (0.52) | <.001^c^ |
| **Group*time^a^** |  |  |
| Intervention*T2 versus control*T2 | 2.28 (0.59) | <.001^c^ |
| Intervention*T1 versus control*T1 | 2.63 (0.51) | <.001^c^ |

^a^ Adjusted gender and economic status

^b^ The difference between the two groups at a significance level of .01 (two-tailed)

^c^ The difference between the two groups at a significance level of .001 (two-tailed)

^d^ SE: Standard Error
